# High salt intake combined with hypertension elevated the risk of primary liver cancer: a prospective cohort study

**DOI:** 10.3389/fonc.2022.916583

**Published:** 2022-08-03

**Authors:** Tong Liu, Qingsong Zhang, Xiaoli Xiao, Yiming Wang, Xiangming Ma, Mengmeng Song, Qi Zhang, Liying Cao, Hanping Shi

**Affiliations:** ^1^ Department of Gastrointestinal Surgery/Clinical Nutrition, Capital Medical University Affiliated Beijing Shijitan Hospital, Beijing, China; ^2^ Beijing International Science and Technology Cooperation Base for Cancer Metabolism and Nutrition, Beijing, China; ^3^ Key Laboratory of Cancer FSMP for State Market Regulation, Beijing, China; ^4^ Department of General Surgery, Kailuan General Hospital, Tangshan, China; ^5^ Department of Gynecology, Aerospace Center Hospital, Beijing, China; ^6^ Department of Hepatological Surgery, Kailuan General Hospital, Tangshan, China

**Keywords:** Hypertension, high-salt intake, liver cancer, joint-effect, prospective

## Abstract

**Background:**

Hypertension and high-salt intake may act synergistically to increase the risk of primary liver cancer (PLC). We prospectively examined the joint effect of hypertension and salt intake on the risk of PLC incidence.

**Methods:**

A total of 92,978 participants were included in the final analyses. The study population was divided into 4 groups according to the presence or absence of hypertension and salt intake. Cox proportional hazards regression models were used to evaluate the association of hypertension and/or high-salt intake with the risk of incident cancers. The CAUSALMED procedure was used to perform the mediation analyses.

**Results:**

During a median follow-up of 12.69 years, a total of 418 incident cancer cases were identified. Hypertension was a risk factor for PLC in women but not in men. High salt intake was associated with an elevated risk of PLC in men. A significant interaction between salt intake and hypertension was found for the risk of PLC (P for interaction=0.045). Compared with Group 1 (hypertension-, high salt intake-), participants in Group 2 (hypertension-, high salt intake+) and Group 4 (hypertension+, high salt intake+) were associated with an elevated risk of PLC with the corresponding multivariate HRs (95%CIs) of 1.73(0.96,3.10) and 1.96(1.09,3.53) respectively. No significant mediation effect was found for the association between hypertension, salt intake and PLC risk.

**Conclusions:**

The combination of high salt intake and hypertension could significantly increase the risk of PLC. It may be reasonable to recommend a low-salt intake to prevent and control the prevalence of PLC and hypertension.

**Trial registration:**

Kailuan study, ChiCTR–TNRC–11001489. Registered 24 August, 2011-Retrospectively registered, https://www.chictr.org.cn/showprojen.aspx?proj=8050

## Introduction

Primary liver cancer (PLC) accounts for 6% and 9% of global cancer incidence and death burden respectively. Liver cancer ranks as the fifth most prevalent cancer in men (554,000 new cases, 8% of total) and the ninth most common cancer in women (228,000 cases, 3% of total), with China accounting for more than half of global incidence and death (1). Established PLC causes include aging, obesity, male sex, alcohol consumption, hepatitis B virus, hepatitis C virus infection, and the consumption of aflatoxin-contaminated food (2–4). However, the occurrence of cancer is complex, with numerous unclear carcinogenic factors. The implementation of preventive analytic strategies relies only on information about carcinogens that affect people in well-established ways.

Hypertension is the key cause of cardiovascular disease leading to premature death (5). Recently, studies proved a positive association between arterial hypertension and the development of several types of cancers including PLC (6–8). It is worth noting that high salt intake, a causal factor of hypertension, has also been proven to be closely related to the occurrence of several types of cancer (9–11). China is a fast-growing and rapidly-aging nation with approximately 200 million hypertensive patients (12), and sodium intake is very high across all regions in China (12 g/person/d) (13). Based on the above findings, hypertension and high salt intake may act synergistically to increase the risk of PLC. However, this hypothesis has not been clarified.

The Kailuan study is a prospective, population-based cohort study with follow-ups conducted every other year. The measurements of blood pressure and the assessment of dietary salt intake provide us with a valuable opportunity to ascertain the joint effect of hypertension and salt intake on the risk of PLC incidence.

## Methods

### Study populations

As described previously (14), the Kailuan Study is a prospective, population-based cohort study conducted in the Kailuan community in Tangshan, China. 101,510 employees or retired workers (81,110 men and 20,400 women, aged 18-98 years) in the Kailuan Company participated in the first health examination between July 2006 and October 2007 and biennial follow-ups. Standardized questionnaire surveys, physical check-ups, clinical assessments, and laboratory tests were conducted for all participants at baseline examination and each follow-up.

In this study, participants were excluded if they: 1) had a history of cancer at baseline (n=377); 2) had missing data or unclear results of systolic blood pressure (SBP, in mmHg), diastolic blood pressure (DBP, in mmHg), or salt level consumption (n=4,611); 3) were without data of other potential confounders including age, sex, body mass index (BMI, in kg/m^2^), total cholesterol (TC, in mmol/L), triglyceride, (TG, in mmol/L), C-reactive protein (CRP, in mg/L), alanine aminotransferase (ALT, in u/L), total bilirubin (TBil, in umol/L), hepatitis B surface antigen (HBsAg), family income of each member, educational background, marital status, smoking status, drinking status, physical activity, sedentary lifestyle, tea consumption, fatty liver, liver cirrhosis, and family history of cancer (n=3,544). Compared to eligible participants, individuals without data of confounders (n=3,544) were relatively younger (51.48 ± 12.45 vs. 50.99 ± 10.10, p for difference<0.05), consisted of more male participants (79.87% vs. 81.21%, p for difference<0.05), and had higher BMI (25.06 ± 3.40 vs. 25.90 ± 3.44, p for difference<0.05). However, no difference was found for the prevalence of HBsAg, drinking and smoking status (all p for difference>0.05).

**Figure 1:**
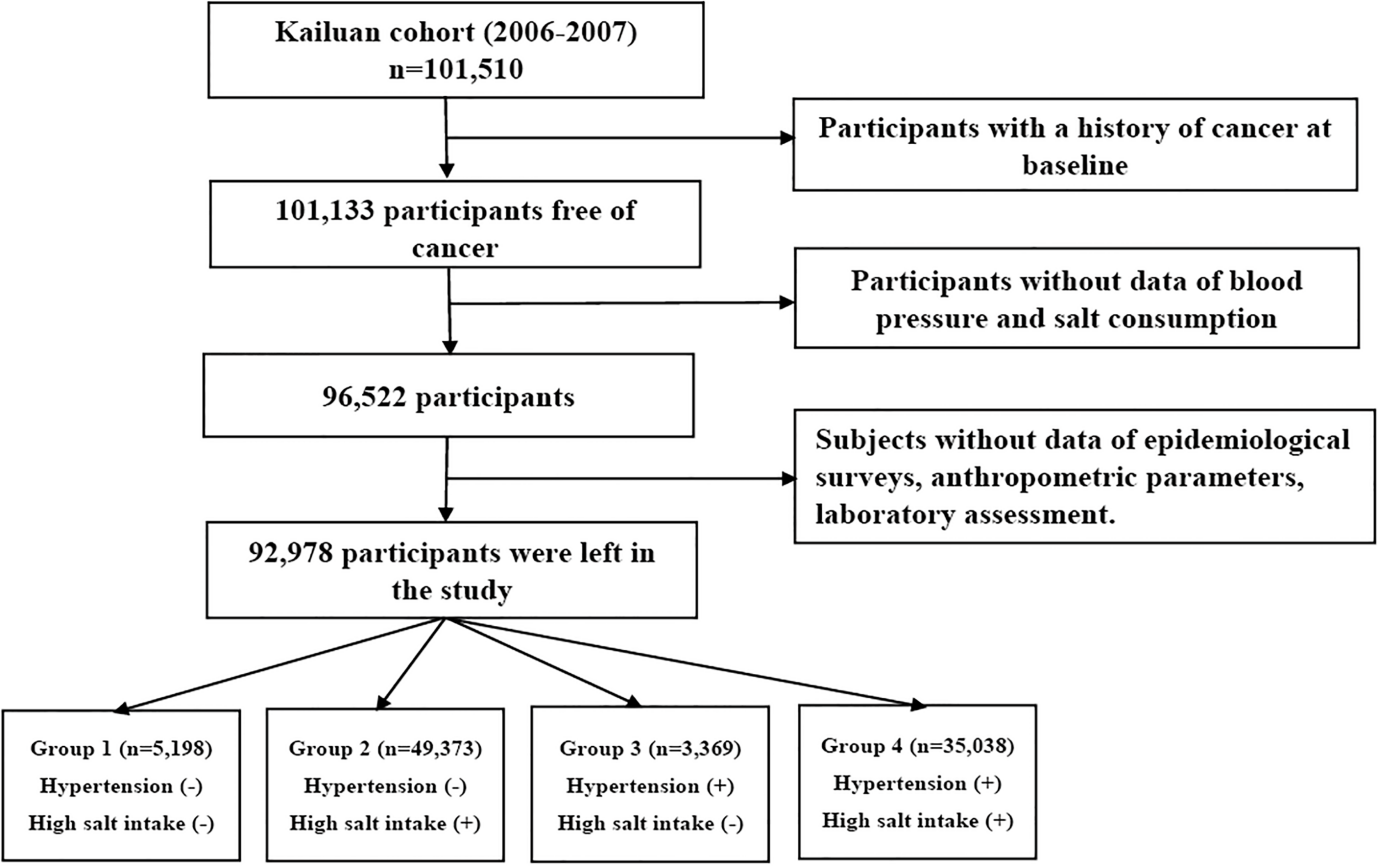
The procedure of participants’ screening.

A total of 92,978 participants were included in the final analyses. The participants’ screening details are shown in **Figure 1**. The protocol for this study was according to the guidelines of the Helsinki Declaration and was approved by the Ethics Committee of Kailuan General Hospital and Beijing Shijitan Hospital. All participants signed written informed consent.

### Assessment of hypertension

Blood pressure was measured in the morning (7-9 am) on the day of the medical examination. After resting for at least 5 minutes, BP was measured twice using a calibrated mercury sphygmomanometer placed on the left arm of each participant in the seated position. BP readings were taken 2 consecutive times, and the average of the 2 readings was used for analysis. Hypertension was defined as having an average of SBP ≥140 mm Hg and/or DBP ≥90 mm Hg, and/or a self-reported history of hypertension, and/or antihypertensive medication use.

### Assessment of salt intake

As described previously (15, 16), information on perceived salt intake was determined *via* a questionnaire survey about regular salt consumption and classified into three categories: <6 g/day (<2400 mg/day sodium intake) for low salt intake; 6-10 g/day (2400–4000 mg/day sodium intake) for intermediate salt intake, and >10 g/day (>4000 mg/day sodium intake) for high salt intake. According to the World Health Organization (WHO) recommendation (<5 g/day), high salt intake was defined as ≥6 g/day in the current study.

In 2012, a validation study was conducted by collecting random spot urine samples from 231 hypertensive participants who did not use any antihypertensive drugs from the Kailuan Study. The results from this study showed that after adjustments were made for age, sex, and blood pressure, a higher perceived salt intake was significantly associated with higher estimated 24-h urinary sodium excretion. The average 24-h urinary sodium excretion was 3745 mg/day, 3930 mg/day, and 3958 mg/day across the three aforementioned perceived salt intake groups (*p* for trend < 0.001) (17).

### Outcome ascertainment

During the study period, incident PLC was collected by 1) tracking participants when they participated in each 2-year routine health examination until 31 December 2019; 2) checking medical records linked with the Tangshan medical insurance system, provincial vital statistics data, and the Kailuan social security information system yearly; and 3) reviewing death certificates from provincial vital statistics offices (PVSO) once a year. The diagnosis of incident cancer was further reviewed by clinical experts. PLC was coded as 22 according to the International Classification of Diseases, Tenth Revision (ICD-10).

### Potential confounders

A face-to-face questionnaire was conducted for each participant to collect information on socioeconomic status, educational background, lifestyle behaviors, personal and familial medical histories. Smoking was defined as having smoked ≥ 1 cigarette/day on average for at least one year. Drinking status was defined as having 100 ml/day of alcoholic beverages for more than one year. Exercise was defined as exercising ≥ 3 times a week lasting ≥ 30 minutes each time. A sedentary lifestyle was defined as sitting for >8 h/day. Tea consumption was defined as drinking tea > 4 times/week.

BMI was classified into normal weight (<24 kg/m^2^), overweight (24.00-27.99 kg/m^2^), or obese (≥ 28 kg/m^2^). The diagnoses of liver cirrhosis and fatty liver were based on the results of abdominal ultrasounds by using real-time ultrasound sonography (PHILIPS HD-15) after each participant fasted for at least 8 hours or were ascertained through medical records linked with the Tangshan medical insurance system.

After an overnight fast (>8 h), 5-ml blood samples were collected from the cubital vein at 7–9 am on the physical examination day. After separating and extracting serum, the same group of laboratory technicians tested the samples using the biochemistry analyzer (Hitachi 7600, Tokyo, Japan) at Kailuan Central Laboratory. Diabetes mellitus was defined as having an FBG level ≥7.0 mmol/L, taking oral hypoglycemic agents or insulin, or having a self-reported history.

### Statistical analysis

The study population was divided into 4 groups according to the presence or absence of hypertension and salt intake: Group 1 (participants without hypertension and high salt intake), Group 2 (participants without hypertension and with high salt intake), Group 3 (participants with hypertension and without high salt intake), and Group 4 (participants with hypertension and high salt intake). Normally distributed variables were described and compared by means ± standard deviations (SD) and one-way analysis of variance (ANOVA). Data with a skewed distribution were described as the median (IQR) and were analyzed by nonparametric tests. Categorical variables were represented as absolute values with percentages and were compared using the chi-square test. Person-years were calculated from the date of the baseline examination to the date of cancer diagnosis, death, or the end of follow-up (31 December 2019), whichever occurred first. Logistic regression analysis was used to examine the relationship between salt intake and hypertension at baseline examination. The proportional hazard assumption was checked using Schoenfeld residual test. Cox proportional hazards analysis was used to estimate the hazard ratios (HRs) and their 95% confidence intervals (CIs) to determine the effect of hypertension and/or high salt intake on the risk of incident PLC. In the subgroup analysis, participants were further stratified by sex, age, HBV infection, drinking and smoking status. The interactions were tested using multiplicative models.

Due to the close association between hypertension and salt-intake, mediation analysis was conducted to explore the relationship between high-salt intake, hypertension, and PLC risk. The mediation analyses based on the variance-covariance matrix and the maximum likelihood technique were carried out using the CAUSALMED procedure. This procedure calculated the total effect, direct effect, and indirect effect.

As a sensitivity analysis, we excluded participants with PLC that had occurred within the first year or the first five years of follow-up to eliminate the possibility of reverse causation. In addition, death (competing risk event) may occur before the diagnosis of PLC. Traditional Cox regression may overestimate the absolute risk in this competing risk setting. We further used the cause-specific hazards (CS) models to calculate HR_CS_ of PLC incidence.

A two-sided P value<0.05 was considered statistically significant. Statistical analyses were performed using the SAS software, version 9.4.

## Results

Among 92,978 participants, the mean (SD) age was 51.48 (12.45) years, with 74,259 (79.87%) men and 18,719 (20.13%) women. The differences in baseline characteristics of the study population are shown in **Table 1**. Significant differences were found in age, levels of TC, TG, ALT, TBil, CRP, and BMI. The percentage of men, per capita income (>800 ¥/month), physical exercise, smoking and drinking status, sedentary lifestyle, tea consumption, fatty liver, diabetes mellitus, and family history of cancer were significantly different among four groups. However, no difference in the prevalence of liver cirrhosis and HBV infection was observed among the 4 prespecified groups.

**Table 1:** Baseline characteristics of the participants stratified by hypertension and salt intake status.

Variables	Group 1	Group 2	Group 3	Group 4	*P*-value
**n**	**5198**	**49373**	**3369**	**35038**	
**Age (year)**	**49.11 ± 12.89**	**48.72 ± 12.38**	**56.95 ± 15.11**	**55.19 ± 11.38**	**<0.001**
**Male (%)**	**3994 (76.84)**	**37367 (75.68)**	**2842 (84.36)**	**30056 (85.78)**	**<0.001**
**TC (%)**	**4.91 ± 1.12**	**4.89 ± 1.08**	**5.09 ± 1.30**	**5.03 ± 1.22**	**<0.001**
**TG (%)**	**1.15 (0.81,1.72)**	**1.18 (0.83,1.76)**	**1.40 (1.00,2.17)**	**1.43 (1.03,2.19)**	**<0.001**
**ALT (%)**	**17.00 (12.00,24.00)**	**18.00 (12.00,24.00)**	**18.00 (12.00,25.00)**	**19.00 (13.20,25.00)**	**<0.001**
**TBil (%)**	**12.30 (9.50,15.50)**	**12.30 (9.90,15.30)**	**12.30 (9.50,15.80)**	**12.10 (9.80,15.10)**	**<0.001**
**CRP (%)**	**0.70 (0.30,1.70)**	**0.70 (0.27,1.80)**	**1.00 (0.40,2.30)**	**0.96 (0.36,2.45)**	**<0.001**
**BMI (%)**	**24.25 ± 3.24**	**24.42 ± 3.35**	**25.84 ± 3.43**	**26.02 ± 3.50**	**<0.001**
**Income of each member (>800 ¥)**	**1365 (26.26)**	**6259 (12.68)**	**1240 (36.81)**	**5711 (16.30)**	**<0.001**
**Physical exercise (%)**	**1365 (26.26)**	**6259 (12.68)**	**1240 (36.810)**	**5711 (16.30)**	**<0.001**
**Smoking status (%)**	**2054 (39.52)**	**15496 (31.39)**	**1333 (39.57)**	**9889 (28.22)**	**<0.001**
**Drinking status (%)**	**1090 (20.97)**	**8016 (16.24)**	**893 (26.51)**	**6657 (19.00)**	**<0.001**
**Sedentary lifestyle (>8 h/d, %)**	**323 (5.59)**	**1687 (3.42)**	**181 (5.37)**	**817 (2.33)**	**<0.001**
**Tea consumption (>4 times/w, %)**	**832 (16.01)**	**4058 (8.22)**	**597 (17.72)**	**3260 (9.30)**	**<0.001**
**Fatty liver (%)**	**1237 (23.80)**	**12287 (24.89)**	**1341 (39.80)**	**14751 (42.10)**	**<0.001**
**Liver cirrhosis (%)**	**7 (0.13)**	**92 (0.19)**	**4 (0.12)**	**55 (0.16)**	**0.566**
**Family history of cancer (%)**	**343 (6.60)**	**1834 (3.71)**	**194 (5.76)**	**1028 (2.93)**	**<0.001**
**HBsAg Seropositive (%)**	**151 (2.90)**	**1424 (2.88)**	**83 (2.46)**	**913 (2.61)**	**0.059**
**Diabetes mellitus (%)**	**316 (6.08)**	**2923 (5.92)**	**400 (11.87)**	**4117 (11.75)**	**<0.0001**

BMI, body mass index; TC, total cholesterol; CRP, C-reactive protein; TG, triglyceride; ALT, alanine aminotransferase; TBil, total bilirubin; HBsAg, hepatitis B surface antigen.

Results presented with bold valued were statistically significant with all p value < 0.05.

**Figure 2:**
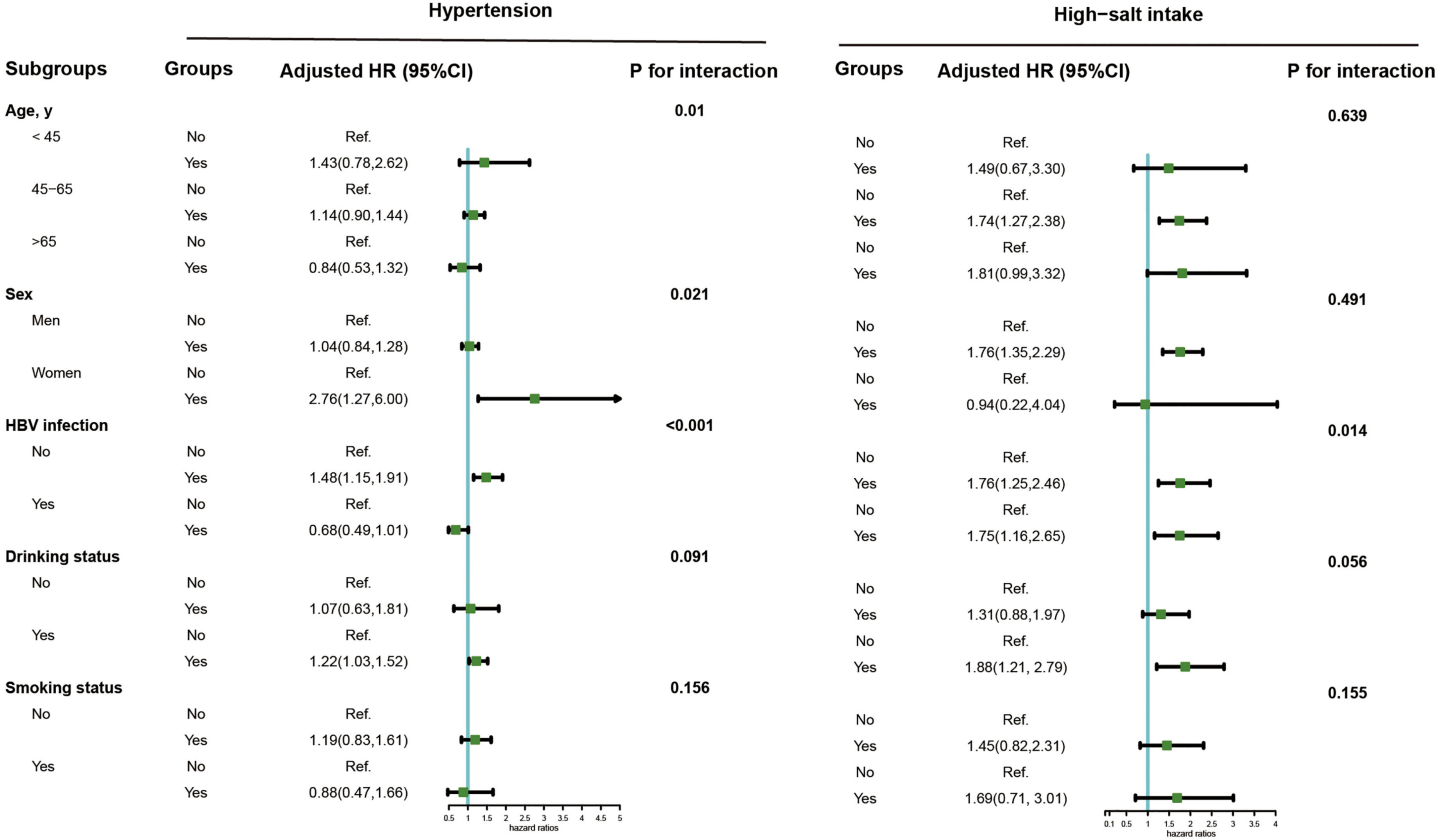
Subgroup analysis of the association of high-salt intake or hypertension with the risk of PLC. Note: Models were adjusted for age (every 10 years), sex, family income, educational background, marital status, BMI, TG, TC, TBiL, ALT, CRP, smoking status, drinking status, physical activity, sedentary lifestyle, tea consumption, diabetes, fatty liver, liver cirrhosis, HBV infection, and family history of cancer except the stratified factors.

During 12.69 years follow-up, a sum of 418 incident cancer cases were identified. The Schoenfeld residual global test was used to verify the proportional hazard assumption, and there was no violation of that hypothesis (*P* = 0.214). **Table 2** presents the HRs (95% CIs) for high salt intake or hypertension as related to the risk of PLC. High salt intake was associated with the risk of PLC incidence. The crude and adjusted HRs (95% CIs) for high vs. low salt intake were 1.76 (1.43, 2.16) and 1.64 (1.33, 2.02) respectively. No significant association between hypertension and PLC risk was found in the multivariate analyses. A significant interaction between salt intake and hypertension was found for PLC risk (*P* for interaction=0.045). **Figure 2** illustrates the association of hypertension or a high salt intake with PLC risk in the subgroup analysis. Significant associations between hypertension and PLC risk were observed among female, HBV seronegative and drinking groups. An elevated risk of high salt intake for developing PLC incidence was found among participants who were middle-aged, male, HBV seronegative, HBV seropositive, and drinker.

**Table 2:** Hazard ratios (HRs) for the association of hypertension or high-salt intake with PLC risk.

Group	Cases/person-years	Crude models		Adjusted models	
		HR (95%CI)	*p*-value	HR (95%CI)	*p*-value
**High-salt intake ^a^**					
**No**	21/104467	Ref.		Ref.	
**Yes**	397/1033045	**1.76 (1.43,2.16)**	<0.001	**1.64 (1.33,2.02)**	<0.001
**Hypertension ^b^**					
**No**	218/677880	**Ref.**		Ref.	
**Yes**	200/459632	**1.34 (1.10,1.62)**	0.003	1.12 (0.92,1.36)	0.233
***P* for interaction ^c^**					0.045

Models were adjusted for age (every 10 years), sex, family income, educational background, marital status, BMI, TG, TC, TBiL, ALT, CRP, smoking status, drinking status, physical activity, sedentary lifestyle, tea consumption, diabetes, fatty liver, liver cirrhosis, HBV infection, and family history of cancer.

a: further adjusted for hypertension.

b: further adjusted for high-salt intake.

c: interaction between high-salt intake and hypertension.

Results presented with bold valued were statistically significant with all p value < 0.05.

**Figure 3:**
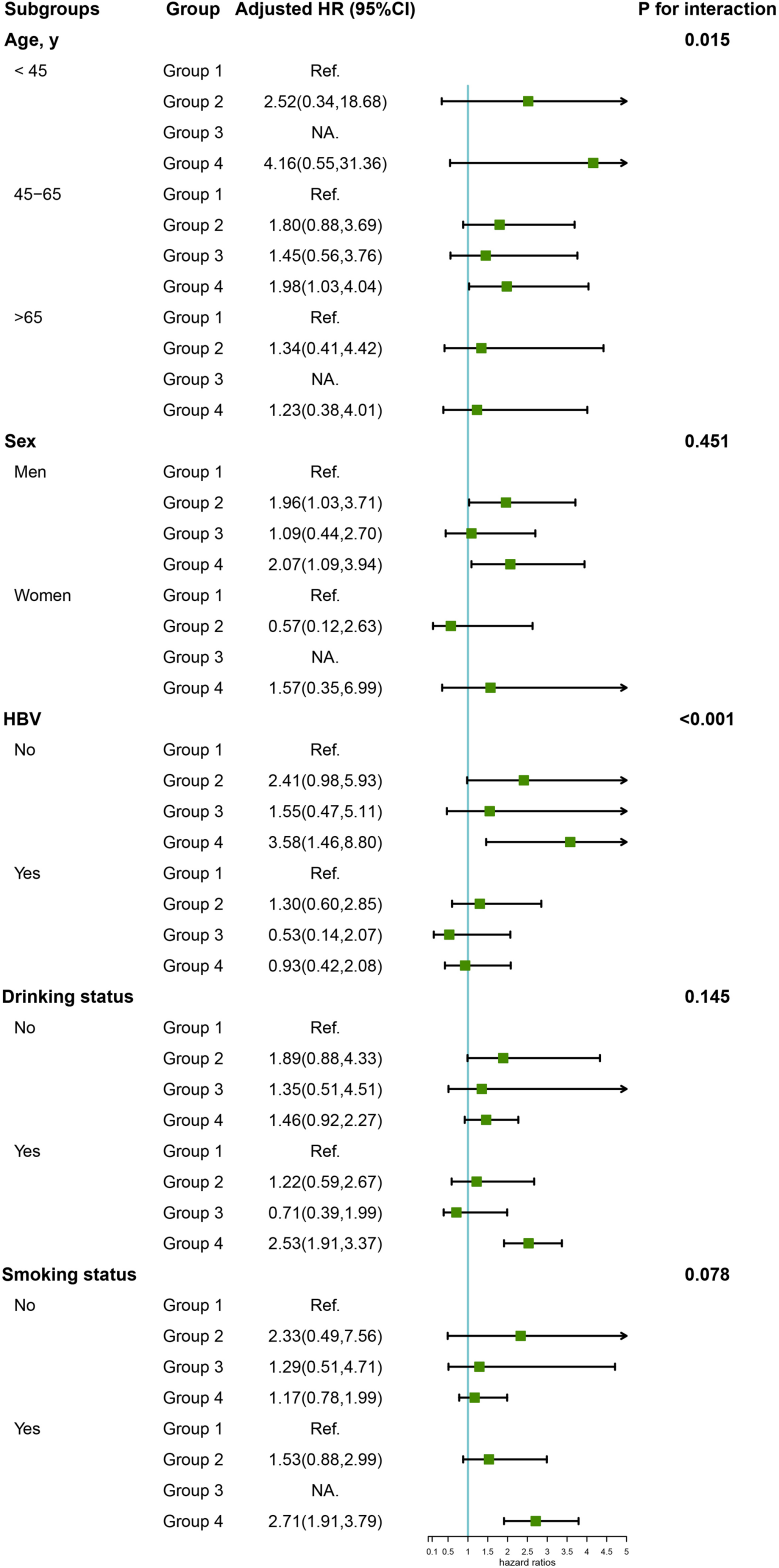
Subgroup analysis of the association of high-salt intake or hypertension with the risk of PLC. Note: Models were adjusted for age (every 10 years), sex, family income, educational background, marital status, BMI, TG, TC, TBiL, ALT, CRP, smoking status, drinking status, physical activity, sedentary lifestyle, tea consumption, diabetes, fatty liver, liver cirrhosis, HBV infection, and family history of cancer except the stratified factors.

**Figure 4:**
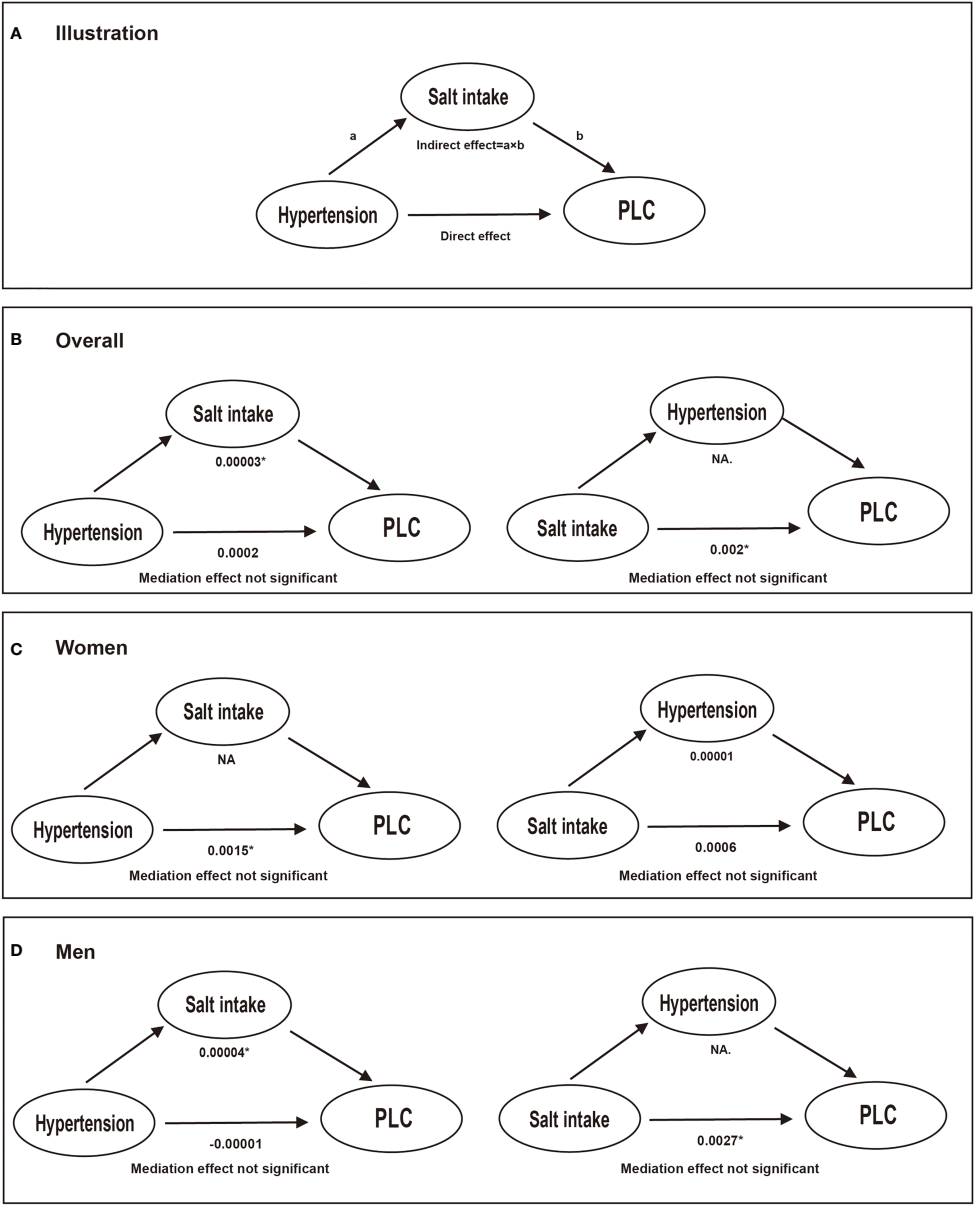
The mediation effect between high-salt intake, hypertension, and liver cancer risk. Models were adjusted for age (every 10 years), sex, family income, educational background, marital status, BMI, TG, TC, TBiL, ALT, CRP, smoking status, drinking status, physical activity, sedentary lifestyle, tea consumption, diabetes, fatty liver, liver cirrhosis, HBV infection, and family history of cancer except the stratified factors. * Values were statistically significant. **(A)** illustration; **(B)** conducted in overall participants; **(C)** conducted in women; **(D)** conducted in men. NA.: not applicable. a: the effect of independent variable on the mediator; b: the effect of the mediator on the outcome.

**Table 3** shows crude and adjusted associations of our main exposure (four categories defined by hypertension and/or high salt intake) with PLC risk. Compared with Group 1 (hypertension-, high salt intake-), participants in Group 2 (hypertension-, high salt intake+) and Group 4 (hypertension+, high salt intake+) were associated with an elevated risk of PLC, with corresponding multivariate HRs (95% CIs) of 1.73 (0.96, 3.10) and 1.96 (1.09, 3.53) respectively. However, there was no significant association between Group 3 (hypertension+, high salt intake-) and PLC risk in the multivariate analyses. Subgroup analyses were further conducted by stratifying participants by sex, age, HBV infection, drinking and smoking status (**Figure 3**). Compared with non-hypertensive and low salt intake participants (Group 1), an elevated risk of PLC was observed among hypertensive participants with high salt intake in the middle-aged, male, HBV seronegative, drinking, and smoking groups. Significant interactions were observed when participants were stratified by age and HBV infection status (P for interaction <0.001). **Figure 4** illustrated the mediation effect between high-salt intake, hypertension, and liver cancer risk. No significant mediation effect was found overall, for both male and female participants.

**Table 3:** Hazard ratios (HRs) for the association of hypertension and high-salt intake with PLC risk.

Group	Cases/person-years	Crude models		Adjusted models	
		HR (95%CI)	*p*-value	HR (95%CI)	*p*-value
**Cox regressions**					
**Group 1**	12/64551	Ref.		Ref.	
**Group 2**	206/613342	**1.82 (1.02,3.26)**	0.044	1.73 (0.96,3.10)	0.068
**Group 3**	9/39916	1.21 (0.51,2.87)	0.670	0.93 (0.39,2.21)	0.871
**Group 4**	191/419703	**2.44 (1.36,4.38)**	0.003	**1.96 (1.09,3.53)**	0.025

Models were adjusted for age (every 10 years), sex, family income, educational background, marital status, BMI, TG, TC, TBiL, ALT, CRP, smoking status, drinking status, physical activity, sedentary lifestyle, tea consumption, diabetes, fatty liver, liver cirrhosis, HBV infection, and family history of cancer.

Results presented with bold valued were statistically significant with all p value < 0.05.

In the sensitivity analysis, after excluding individuals who had PLC within the first year (n=40) or the first five years of follow-up (n=164), the association of hypertension and high salt intake with the risk of PLC incidence remained significant in the multivariate analysis (**Table 4**). During the follow-up, 9123 cancer unrelated deaths (competing risk event) occurred before the diagnosis of PLC. In the competing risk analyses (CS models), the association was attenuated but still significant in the multivariate models.

**Table 4:** Sensitivity analyses after excluding participants who occurred PLC within the 1^st^ year.

Group	Cases/person-years	Adjusted models	
		HR (95%CI)	*p*-value
**Excluded PLC occurred within the 1st year of follow-up**			
** Group 1**	9/64550	**Ref.**	
** Group 2**	186/613332	**2.08(1.07,4.09)**	**0.032**
** Group 3**	8/39915	1.11(0.43,2.89)	0.825
** Group 4**	175/419698	**2.41(1.23,4.73)**	**0.011**
**Excluded PLC occurred within the first 5 years of follow-up**			
** Group 1**	6/64541	**Ref.**	
** Group 2**	126/613122	2.03(0.97,4.01)	0.077
** Group 3**	6/39909	1.17(0.47,2.92)	0.711
** Group 4**	116/419462	**2.49(1.26,4.81)**	**0.005**
**Cause-specific hazards models**			
** Group 1**	12/64551	Ref.	
** Group 2**	206/613342	1.69(0.92,3.05)	0.121
** Group 3**	9/39916	0.89(0.35,2.14)	0.891
** Group 4**	191/419703	**1.93(1.06,3.49)**	0.022

Models were adjusted for age (every 10 years), sex, family income, educational background, marital status, BMI, TG, TC, TBiL, ALT, CRP, smoking status, drinking status, physical activity, sedentary lifestyle, tea consumption, diabetes, fatty liver, liver cirrhosis, HBV infection, and family history of cancer.

Results presented with bold valued were statistically significant with all p value < 0.05.

## Discussion

In this large, population-based prospective cohort study, when comparing hypertensive and non-hypertensive participants with low and high-salt diets, we found that hypertension and high salt intake act independently and synergistically to increase the risk of PLC. This significant association was further observed among middle-aged, male, HBV seronegative, drinking and smoking individuals. To the best of our knowledge, this is the first study to evaluate the impact of the joint effect of hypertension and high salt intake on the risk of PLC worldwide.

In the current study, we found that hypertension combined with high-salt intake was associated with an elevated risk of PLC, and hypertension alone also increased the risk of PLC incidence among women, which was in line with previous studies. A large prospective cohort study found that mid-BP ([SBP + DBP]/2) was positively associated with incident PLC (8). GD Batty et al. found that both SBP and DBP were positively related to death from PLC in a large-scale prospective investigation of male government employees in the Whitehall study (18). Although studies focused on the association between hypertension and PLC are limited, several large prospective studies have suggested that elevated blood pressure is associated with an increased risk of incident kidney (19, 20), prostate (7), bladder (21), pancreatic (22), endometrial (23), and breast cancer (24).

Additionally, our results suggested that participants on a high-salt diet exhibited an elevated risk of liver cancer. Similar results were also obtained among non-hypertensive participants who were on a high-salt diet. A population-based cohort study found that high salt intake was associated with an increased risk of liver cancer during an 11-year follow-up among 97,006 Chinese adults (25). Maud Rizk et al. found that the consumption of sodium elevated the risk of hepatocellular carcinoma (HCC) in a case-control study.

We first found that high salt consumption and hypertension interacted with the development of liver cancer. Compared with non-hypertensive and low-salt diet participants, hypertensive participants on a high-salt diet had a 1.9-fold elevated risk of PLC incidence. The hypertension-salt interaction has important clinical implications because the combined effects of both can be minimized, if not avoided, by reducing salt intake. Excessive dietary salt intake is associated with an increased risk for hypertension (26, 27); on the other hand, the results from a meta-analysis of some interventional studies have shown that a reduction in dietary salt intake leads to a significant reduction in blood pressure especially in hypertensive patients, and to a lesser extent in normotensive patients (28). Future epidemiologic and experimental studies will be needed to explore whether reducing salt intake can prevent the occurrence of cancer.

The underlying mechanism by which high salt intake combined with hypertension increased the risk of incident cancer remains uncertain. The tumorigenic effect of a high-salt diet may include the following aspects: 1) High concentrations of NaCl will increase DNA breaks both in cell cultures and *in vivo (* 29). As long as NaCl is kept at a high level, the breaks remain elevated and will be repaired quickly when the concentration decreases (30), which damages DNA and impairs its repair. 2) Studies on mice have shown that sodium chloride promotes tissue inflammation and autoimmune diseases by playing a role in the modulation of the immune system (31, 32). 3) The high-salt diet also includes the consumption of processed foods such as ham and pickles that are rich in sodium nitrite (NaNO_2_). Long-term exposure to sodium nitrite has been found to be carcinogenic in multiple organs (33). In addition, the tumorigenic effects of hypertension may include the following aspects: 1) cancer and hypertension may share a common pathophysiological pathway mediated by adipose tissue, which may lead to chronic inflammation and further increase the risk of cancer and hypertension (34, 35). 2) Another possible explanation is that hypertension may increase the risk of cancer by blocking and altering apoptosis and thus affecting the regulation of cell turnover (36).

The main merit in the current study is that it has offered a unique perspective on the potential association of the combination of high salt intake and hypertension with PLC risk. Furthermore, this study addresses a wide range of assessments of potential confounders, including lifestyle behaviors and a history of cancer-associated diseases. Finally, cancer cases were obtained through inspections of the Tangshan medical insurance system and Kailuan social security system, which recorded all health information of participants. Using this method, the follow-up rate was almost 100% in the current study.

The main limitation of this study is that salt intake is assessed based on self-reports, even though the field staff is well-trained in obtaining information. As we described previously, a significant dose-response relationship between perceived salt intake and urinary sodium excretion was further found in a validation study. Other limitations are also worth noting. We did not collect detailed information on the hepatitis C virus (HCV), which prevents us from more accurately assessing confounding factors. However, the prevalence of hepatitis C core antibodies in China is only 0.43%, and may have little impact on the results. Furthermore, due to different dietary patterns and genetic factors, the dietary salt intake of Western populations is much lower than that of the Chinese population (37). Thus, extrapolated results might not be an accurate description of the Western population. Lastly, although we used the average BP readings which were taken 2 consecutive times, misclassification of hypertension may still exist in the current study.

## Conclusions

In summary, the results of this prospective cohort study showed that the combination of high salt intake and hypertension could significantly increase the risk of PLC. It may be reasonable to recommend low salt intake to prevent and control the prevalence of cancer and hypertension. This suggestion may be more applicable to Asian populations who consume more salt than Western populations.

## Data availability statement

The raw data supporting the conclusions of this article will be made available by the authors, without undue reservation.

## Ethics statement

The studies involving human participants were reviewed and approved by Beijing Shijitan Hospital and Kailuan General Hospital. The patients/participants provided their written informed consent to participate in this study.

## Author contributions

All authors have read and approved the manuscript. TL: Methodology, Software, Writing- Original draft preparation, QSZ Writing- Reviewing and Editing. XLX Writing- Reviewing and Editing. YMW: Supervision, Validation. XMM: Software. MMS: Writing- Reviewing and Editing. QZ: Writing- Reviewing and Editing. LYC: Conceptualization, Supervision. HPS: Conceptualization, Supervision, Validation, Resources.

## Funding

This work was financially supported by the National Key Research and Development Program (2017YFC1309200) and the Beijing Municipal Science and Technology Commission (SCW2018-06) to Dr. Hanping Shi.

## Acknowledgments

We thank all the staff and participants of the Kailuan study for their important contributions.

## Conflict of interest

The authors declare that the research was conducted in the absence of any commercial or financial relationships that could be construed as a potential conflict of interest.

## Publisher’s note

All claims expressed in this article are solely those of the authors and do not necessarily represent those of their affiliated organizations, or those of the publisher, the editors and the reviewers. Any product that may be evaluated in this article, or claim that may be made by its manufacturer, is not guaranteed or endorsed by the publisher.
